# A Graph Localization Approach for Underwater Sensor Networks to Assist a Diver in Distress †

**DOI:** 10.3390/s21041306

**Published:** 2021-02-11

**Authors:** Roee Diamant, Roberto Francescon

**Affiliations:** 1 Hatter Department of Marine Technologies, University of Haifa, Haifa 3498838, Israel; 2 Department of Information Engineering, University of Padova, Via Gradenigo 6/B, 35141 Padova, Italy; frances1@dei.unipd.it

**Keywords:** SOS transmission, underwater acoustic communication, underwater acoustic networks, graph localization, underwater localization

## Abstract

In this paper, we focus on the problem of locating a scuba diver in distress using a sensor network. Without GPS reception, submerged divers in distress will transmit SOS messages using underwater acoustic communication. The study goal is to enable the quick and reliable location of a diver in distress by his fellow scuba divers. To this purpose, we propose a distributed scheme that relies on the propagation delay information of these acoustic SOS messages in the scuba divers’ network to yield a range and bearing evaluation to the diver in distress by any neighboring diver. We formalize the task as a non-convex, multi-objective graph localization constraint optimization problem. The solution finds the best configuration of the nodes’ graph under constraints in the form of upper and lower bounds derived from the inter-connections between the graph nodes/divers. Considering the need to rapidly propagate the SOS information, we flood the network with the SOS packet, while also using rateless coding to leverage information from colliding packets, and to utilize time instances when collisions occur for propagation delay evaluation. Numerical results show a localization accuracy on the order of a few meters, which contributes to quickly locating the diver in distress. Similar results were demonstrated in a controlled experiment in a water tank, and by playback data from a sea experiment for five network topologies.

## 1. Introduction

In the last decade, ocean exploration has considerably increased, as has the involvement of human divers performing maintenance work and site surveys. In the harsh underwater environment, these activities pose a great risk to divers’ safety. However, due to the constraints of their work, divers may sometimes find themselves in need of assistance when they are far away from their fellow divers. In such cases, modern diving systems include a way to acoustically broadcast an SOS message, e.g., [1]. These messages include the ID of the diver in distress and propagate well in the divers’ network. However, without a way for fellow divers to identify the location of the diver in distress, these systems cannot provide immediate help.

In the absence of GPS reception, underwater localization mostly involves measuring the range and/or bearing to the acoustic source. While common solutions use trilateration from multiple anchor nodes or utilize the relative motion between the receiver and transmitter [2], localization of the source of an SOS packet requires a different approach. First, the need for a rapid response does not allow a centralized approach, such as trilateration. Second, due to the irregular motion of scuba divers, especially those working underwater for long time periods of several hours, relying on self-navigation means for localization-such as inertial sensors, is not practical. Third, since the accurate pre-deployment of beacons is challenging in complicated environments, such as harbors where most underwater works occur, it is impractical to use anchor nodes in the localization solution. Instead, considering the likely sparsity of the divers’ network, a graph localization (GL) approach is a better fit.

Solving through GL refers to the construction of a graph from links between sensor nodes and the positioning of the nodes within the graph, by turning and stretching the graph to best fit a utility function under constraints on the inter-connections between the nodes. Many formalized and heuristic solutions, including our preliminary work in [3], are offered for GL with the aim of encompassing ranges for all of the links in the graph. However, for underwater acoustic networks—where time-of-flight (ToF) measurements are often noisy with many outliers and where the network’s graph is sparse—this objective may degrade the localization performance. Considering this problem, we propose a novel GL formalization, whose utility not only fits the structure of the formed graph to ToF measurements, but also aims to minimize the impact of outliers while setting constraints on the range between the graph’s links. The result is a constraint multi-objective optimization problem that is solved by semi-definite programming. To further manage infeasibilities due to outliers, we present a relaxed convex optimization problem. Considering the need to quickly propagate the SOS message, we flood the SOS packet throughout the network. The expected collisions between the packets are handled through rateless coding. The capability to identify erasure symbol indicators in rateless coding also allows a ToF estimation for colliding packets. This way, no cooperation in scheduling is required between the network’s nodes, and the delay from the time of the SOS transmission to the time of the localization decision reached at each node is obtained quickly. Numerical simulations show that the resulting localization accuracy is on the order of a few meters, which is acceptable for the purpose of approaching the diver in distress. The results were obtained by conducting a controlled experiment in a water tank, and using data from a sea experiment for five network topologies.

To summarize, compared to the state-of-the-art, our contribution and novelty are four-fold:

- A graph localization solution that includes upper and lower bounds—not just as regards the constraints of the graph optimization problem (as other methods propose)—but also in its utility. As our results show, minimizing the difference between the upper and lower bounds leads to more accurate location identification.
- While previous methods only derive the graph localization problem, in cases where no feasible solution is found, our approach also offers a fallback in the form of a relaxed optimization problem. This is important since, due to the typical erroneous time-of-flight measurement and the sparse graph in underwater acoustic networks, rigid constraints may not allow a feasible solution.
- In contrast to current approaches that utilize time-of-flight information for the graph’s link only for correctly received packets, our approach also offers a way to extract such information from colliding packets. This is important because, as a result of flooding, for the SOS packets across the network, many collisions are expected. We offer this capability using a novel scheme that derives the time-of-flight from the erasure indicators of a rateless code. Hence, the availability of the localization solution is improved, thereby decreasing the delay from the SOS transmission time to its source localization.
- To the best of our knowledge, ours is the first formalized graph localization approach for underwater acoustic networks. Since underwater acoustic communication has significant differences compared to radio frequency terrestrial networks, this contribution is not just in terms of the considered application, but also requires the modification of the localization solution. This is reflected by the choice of the upper and lower bounds in the graph localization, in the SOS decoding protocol, and in the expected errors of the time-of-flight estimation. We have included sea trial results to support our claims.

The remainder of this paper is organized as follows. In Section 2, we present the state-of-the-art for underwater localization in the context of communication networks. In Section 3, we introduce our system’s model and assumptions. Section 4 describes the details of our proposed SOS localization algorithm. In Section 5, we share results from numerical simulations and from real environments. Conclusions are drawn in Section 6. The main notations used in this paper are summarized in Table 1.

## 2. State-of-the-Art for Underwater Network Localization

Underwater acoustic localization is a challenging task: Range estimations are affected by non-line-of-sight reflections, the transmitter–receiver pairs are often not time-synchronized, and the sound speed in water is not always known. Moreover, due to motion, a node hardly obtains range measurements to at least three anchor nodes while remaining at a fixed location. In [2,4,5], comprehensive surveys are presented for localization methods using underwater acoustics. The available methods can be categorized as passive localization, where the node-to-be-localized does not transmit signals, and active localization, where the node cooperates with other nodes via two-way signal transmissions to estimate its range and bearing to beacons through triangulation.

In the context of underwater sensor networks, a main challenge is the lack of a sufficient number of beacons to resolve localization ambiguities. This so-called under-ranked localization problem is solved by either considering already localized nodes as new, second degree, anchors [6]; or by utilizing the nodes’ motion. In [7], localization using a single beacon is achieved by tracking the node’s range over time. In [8], environmental information is utilized to estimate the location of the node within a map of likelihood values obtained by matching the measured channel to a propagation model. Similarly, reference [9] ranks localization ambiguities by their match to the signal’s power spectra. However, performance quickly degrades when the environmental information is even slightly mismatched. For indoor localization, the authors of [10] used side information in the form of detection ranges to resolve the likelihood ambiguity problem, and template matching to a pre-measured signal strength is offered in [11]. However, more leverage can be gained by utilizing the possible relationships among the sensor nodes.

As evaluating the location of the diver in distress involves a sparse network, a solution may be found in GL tools, where nodes reflect vertices and ranges between nodes as non-directed edges. The localization solution is found by shifting and rotating the nodes within the graph so as to optimize a utility function, while setting the measured ranges between the nodes as problem constraints [12]. A complete analysis of the GL problem is given in [13], where the theory behind GL is expounded along with an optimal algorithm for solving the problem.

The most common GL approach is Multidimensional Scaling (MDS) [14], which finds a lower dimensional space that represents the graph by eigenvalue–decomposition to translate pairwise distances between nodes into an *n*-dimensional map. MDS variants allow for the setting of bounds on inter-link ranges and the filtering of solutions to mitigate error propagation from ranging inaccuracies [15,16,17]. Other approaches formalize the graph localization under proximity constraints in cases where the graph’s connections are known [18], by manifold learning [19] and as a weighted least-square solution that ranks the feasibility of the node’s relative locations [20]. However, the desire of the above methods to encompass ranges for all of the links in the graph degrades the performance for sparse networks, such as in underwater localization.

When the network graph is sparse and information on distances for some links is missing, the MDS algorithm can be modified to allow under-ranked graphs by applying constraint optimization on the Euclidean distance matrix [21]. The missing data for the distances can also be completed by applying a principle component analysis and decomposing the Euclidian matrix into observable and partly observable parts [22]. An alternative to MDS is the graph embedding approach [23], which makes use of a kernel function to map received signal strength information (RSSI) to the physical coordinates’ space. The solution for the optimization problem is efficiently obtained in [18], using a convex simplification solved by Semi-Definite Programming (SDP) under constraints set by the graph connectivity information. To achieve higher accuracy, reference [19] uses manifold learning to calculate the mapping function between the signal strength measures’ space and the coordinates’ space, but relies on periodic updates from beacons. A modification proposed in [20] solves a regression problem after an optimization of the kernel function used for the initial mapping, and uses either RSSI or ToF. The proposed solutions mostly focus on the GL optimization conversion, and do not directly consider the problem of GL in a highly sparse network, where both beacon information and ranging measurements are scarce. However, regarding the problem of finding the location of the diver in distress in a multi-obstacle-commonly-shallow-water environment, this is the network’s likely structure.

## 3. System Model

Our system includes a set N of *N* nodes, arranged as a non-directed graph. Due to the many obstacles expected in the diver’s working area, the graph’s connectivity is expected to be sparse. Each node is equipped with an underwater acoustic modem, which enables the scheduled transmission of packets within a network (using, e.g., the scheduling protocol in [24].

While a more accurate solution for underwater acoustic localization can be obtained by utilizing angle-of-arrival (AoA) measurements in the form of an ultra-short-baseline solution [25], this involves either a rigid hydrophone 3D array, which is hard to employ over the body of a scuba diver, or a 3D vector sensor, which is extremely expensive and is limited to low carrier frequencies. Instead, for a practical solution, we consider an omnidirectional-single-transceiver modem. We assume the modems are time-synchronized by an underlying process (e.g., [26]), their hardware/software delays in relaying a packet can be accurately estimated (e.g., [27]), and can estimate the range to the transmitting node by measuring the ToF of a packet and the effective sound speed in the link (using, e.g., [28]). We further assume access to the modem’s physical layer, such that reliability measures of decoded symbols are available. These can be, for example, the log-likelihood ratio of a certain symbol to belong to the decoded hypothesis, the symbol’s signal-to-noise ratio, or the normalized matched filter’s output of the symbol. All of the above assumed features of the acoustic modem are available in the EvoLogics 18-34 software-defined modem [29], which we use in our experiments.

Our algorithm is performed distributively at all the network nodes. Without the loss of generality, at a certain time instance, node s∈N issues an SOS message. This node is referred to as the *SOS source*, i.e., the diver in distress. Our solution works distributively at each node i∈N to evaluate the position of node *s*, $p_{s}\left(i\right)$, within a Cartesian coordination system relative to node *i*, which, in turn, is set at (0,0). To simplify terms, in the following, we refer to node *i* as the *assisting node*, but recall that any node in the network that aims to assist the diver in distress would be called an assisting node. To evaluate $p_{s}\left(i\right)$, the assisting node draws from distance estimates between the graph’s edges and from range and bearing estimates to its one-hop neighbors (using, e.g., the method in [30]).

Our algorithm avoids using anchor nodes or surface vessels, and the SOS source localization is performed distributively at each diver’s modem. Our solution is thus practical for the considered applications of underwater construction works and underwater manned surveys where nodes are always submerged and cannot track their own position. As a result, since errors in time-of-flight measurement are proportional to the travel distance, as are the differences between the actual distance and upper and lower bounds for missing communication links, when the scuba divers are close to each other, the accuracy of the localization solution improves. Relying on depth information provided by the SOS source, we aim for two-dimensional space localization. An accurate and timely evaluation of the SOS source’s location will allow the assisting node to promptly respond to the distress call.

## 4. Proposed SOS Localization Algorithm

### 4.1. Key Idea

**Figure 1:**
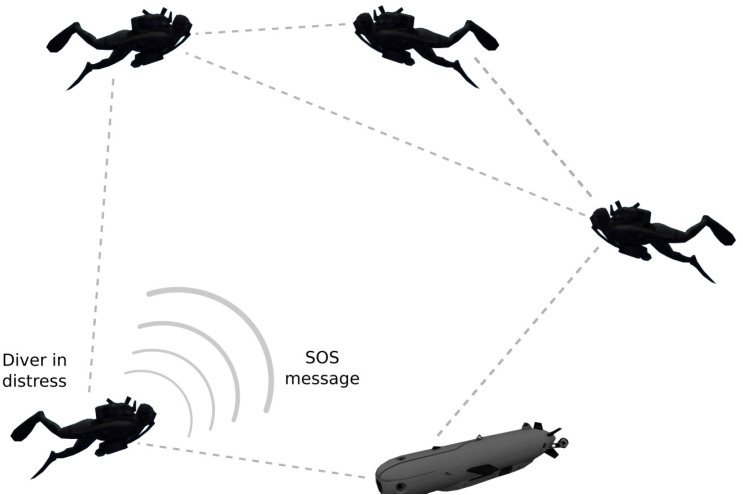
Illustration of system setting.

Referring to the illustration in Figure 1, our solution to evaluate the location of the SOS source, relative to that of the assisting node, is based on propagating the SOS packet across the network while estimating the distances along the graph’s edges. We then use these distances as constraints to project the distance matrix into a feasible relative geographical map. The distance within the communication link is obtained distributively by each node in the graph—either directly by detecting acoustic transmissions of one-hop neighbor nodes, or indirectly by piggy-backing such information on the relayed SOS packet.

In contrast to solutions such as [31], which utilize packets’ known emission intervals to solve localization ambiguities, we look for the fastest way to propagate the SOS and thus choose to flood the messages in a non-organized fashion. During flooding, upon receiving a packet, each node immediately relays the SOS packet until a timeout occurs. To compensate for the expected packet collisions during this flooding, we use rateless channel coding [32], which also assists in the estimating of ToF for the interfering packets. Our algorithm works on several levels: SOS propagation, propagation delay estimation for incoming packets, and per-node localization of the SOS source. The details of each level are presented below, starting from the final location and down to the SOS propagation.

### 4.2. Position of the SOS Source

To evaluate $p_{s}\left(i\right)$, we use a matrix of evaluated distances, D(i), as input between the graph’s edges as observed by node *i*. For a pair of nodes n,m∈N, entry $D_{n,m}\left(i\right)=c\cdot T_{n,m}^{\mathrm{pd}}\left(i\right)$, where *c* is the sound speed in water and $T_{n,m}^{\mathrm{pd}}\left(i\right)$ is the ToF as evaluated directly or indirectly (details of how to obtain D(i) are provided below). If node *i* cannot evaluate the delay in a link (n,m), we set $D_{n,m}\left(i\right)=0$.

To estimate $p_{s}\left(i\right),\;j\in N$, we offer two alternatives. The first, considered as a benchmark, is the multidimensional scaling (MDS) algorithm, which relies on an eigenvalue decomposition of D(i) [14]. Given D(i), MDS provides a map representation of the graph. To project this map representation into the UTM coordination system relative to the assisting node *i*, we shift the MDS solution to fit the node *i* in coordinates (0,0), and rotate the shifted map to fit the relative coordinates on the evaluated node *a*. This process is explained in detail in the following Section 4.2.2. While MDS provides a feasible solution, it does not allow an initial guess and may therefore fall into a local minimum of its loss function, which may lead to large errors. In addition, MDS does not allow for the utilization of prior information, such as upper and lower bounds on the information in D(i). We therefore offer a second alternative, referred to as *conditional GL* (CGL), whose output is the solution of an optimization problem that takes into account some upper and lower bounds on D(i).

#### 4.2.1. Finding the Coordinate Representation of the Graph

For each node pair (n,m),n,m∈N, the CGL algorithm sets an upper bound and a lower bound for the Euclidean distance, $U_{n,m}\left(i\right)$ and $L_{n,m}\left(i\right)$, respectively. The former uses the accumulated propagation delay between non-neighbor nodes along the packet’s route until node *i*. In contrast, the latter is set according to the maximal delay in the packet’s route. That is, $L_{n,m}\left(i\right)=c\cdot \max\left\{T_{p,q}^{\mathrm{pd}}\right\}-\Lambda ,\;\left(p,q\right)\in M_{n,m}$, where $M_{n,m}$ is the shortest pass route from node *n* to node *m*. Parameter Λ is the maximum error of the estimated distance, and is modeled as a function of the maximum expected delay spread in the channel, the ambiguity of the modem’s immanent time delay, and the sound speed in water. Since the lower bound is loose, we introduce a scale parameter, $\rho_{L}$, which allows flexibility.

The CGL solution is found by matching the upper and lower bounds with the measured distances in D(i). Formally, let A be a set of nodes whose locations $p_{a}\left(i\right),\;a\in A$ relative to node *i* are estimated by the assisting node via range and bearing estimations (Note that A can hold even one node), we obtain the estimated nodes’ locations, $\hat{P}\left(i\right)=\{{\hat{p}}_{s}\left(i\right),\ldots ,{\hat{p}}_{N}\left(i\right)\}$, by solving the following optimization problem:

(1a) $$\begin{array}{cc} \hat{P}(i)=\; & \underset{\tilde{P}(i)}{\mathrm{argmin}}\sum_{\left(n,m\right)|D_{n,m}>0}\{\left[\left|\right|{\tilde{p}}_{n}\left(i\right)-{\tilde{p}}_{m}\left(i\right){\left|\right|}_{2}D_{n,m}\left(i\right)\right],\;|\rho_{L}\cdot L_{n,m}\left(i\right)-U_{n,m}\left(i\right)\left|\right\}\; \end{array}$$

(1b) $$\begin{array}{cc} s.t.\;\;\; & \left|\right|{\tilde{p}}_{n}\left(i\right)-{\tilde{p}}_{m}{\left(i\right)||}_{2}\leq U_{n,m}\left(i\right)\;\forall n,m|D_{n,m}>0\;, \end{array}$$

(1c) $$\begin{array}{cc} \;\;\; & \left|\right|{\tilde{p}}_{n}\left(i\right)-{\tilde{p}}_{m}{\left(i\right)||}_{2}\geq \rho_{L}\cdot L_{n,m}\left(i\right)\;\forall n,m|D_{n,m}>0\;. \end{array}$$

(1d) $$\begin{array}{cc} \;\;\; & L_{n,m}\left(i\right)\leq \rho_{L}\cdot L_{n,m}\left(i\right)\leq U_{n,m}\left(i\right)\forall n,m\;. \end{array}$$

(1e) $$\begin{array}{cc} \;\;\; & p_{n}\left(i\right)-\Lambda \leq {\tilde{p}}_{a}\left(i\right)\leq p_{a}\left(i\right)+\Lambda ,\;a\in A\;. \end{array}$$

(1f) $$\begin{array}{cc} \;\;\; & {\tilde{p}}_{i}\left(i\right)=\left(0,0\right)\;. \end{array}$$

Problem (1) is multi-objective with two related objectives. The first aims to minimize the difference between the Euclidean distances of the evaluated locations and the measured distances, and the second guides the solution that minimizes the difference between the lower bound and upper bounds. As a result, the solution would not only seek for a minima in terms of the Euclidean difference between the measured delays and the SOS source location, but would also lean towards a solution with tight constraints, thereby reducing the impact of outliers in the time-of-flight measurements. Note that the objective function avoids the use of node pairs with no connecting route. The problem is constraint, such that the upper and lower bounds on the estimated coordinates fit the evaluated distance matrix D(i) (Constraint (1b) and (1c)), and to limit the range for the lower bound scale factor $\rho_{L}$ (Constraint (1d)). Constraint (1e) fixes the estimated coordinates ${\tilde{p}}_{a}\left(i\right),\;a\in A$ to their known relative locations, while providing leverage to the size of the error ambiguity Λ to allow a feasible solution. Finally, constraint (1f) fixes the coordinates of the assisting node to the location of the assisting node at (0,0).

Note that, in the case of $L_{n,m}\left(i\right)=0$, problem (1) is convex and can be solved via a combination of semi-definite programming (cf. [18]) and multi-objective optimization (e.g., [33]). However, for a non-zero lower bound, (1) is non-convex and its convergence to a global minimum is not guaranteed. Considering this problem, we use the solution of the benchmark MDS algorithm as an initial guess, and solve (1) via quadratic programming. However, in cases where the MDS provides a bad initial estimate or when the estimated distance matrix D(i) includes a large error, the algorithm may not find a feasible solution. We therefore also consider a more relaxed version of (1) that avoids imposing the locations of the nodes in A:

(2a) $$\begin{array}{cc} \hat{P}(i)=\; & \underset{\tilde{P}(i)}{\mathrm{argmin}}\sum_{\left(n,m\right)|D_{n,m}>0}\left[\left|\right|{\tilde{p}}_{n}\left(i\right)-{\tilde{p}}_{m}\left(i\right){\left|\right|}_{2}D_{n,m}\left(i\right)\right]+\left|\rho_{L}\cdot L_{n,m}\left(i\right)-U_{n,m}\left(i\right)\right|\; \end{array}$$

(2b) $$\begin{array}{cc} s.t.\;\;\; & \left|\right|{\tilde{p}}_{n}\left(i\right)-{\tilde{p}}_{m}{\left(i\right)||}_{2}\leq U_{n,m}\left(i\right)\;\forall n,m|D_{n,m}>0\;, \end{array}$$

(2c) $$\begin{array}{cc} \; & \left|\right|{\tilde{p}}_{n}\left(i\right)-{\tilde{p}}_{m}{\left(i\right)||}_{2}\geq \rho_{L}\cdot L_{n,m}\left(i\right)\;\forall n,m|D_{n,m}>0\;. \end{array}$$

(2d) $$\begin{array}{cc} \; & L_{n,m}\left(i\right)\leq \rho_{L}\cdot L_{n,m}\left(i\right)\leq U_{n,m}\left(i\right)\forall n,m\;, \end{array}$$

and directly uses the MDS output as an initial solution, i.e., its non-shifted map representation. Problem (2) is distinctively easier than (1), and is a uni-objective constraint convex optimization problem that holds a global minima. We obtain its solution by the simplex method. We distinguish problem (1) from problem (2) by referring to the former as *direct optimization* and to the latter as *relaxed optimization*.

#### 4.2.2. Matching of Coordinates

Both the initial MDS solution for direct optimization (1) and the solution for relaxed optimization (2) require matching the resulting map representation to the coordination system of the assisting node. We now discuss the process of this correction.

To project the solution of the MDS or of the relaxed optimization into a coordination system relative to the assisting node *i*, we perform shifting through

(3) $$\beta =p_{i}\left(i\right)-{\hat{p}}_{i}\left(i\right)\;,$$

and the estimated coordinates of a node j∈N are shifted by

(4) $${\hat{p}}_{j}^{s}\left(i\right)={p^{\mathrm{init}}}_{j}\left(i\right)-\beta \;,$$

where ${p^{\mathrm{init}}}_{j}\left(i\right)$ is either the output of the MDS (to solve (1)) or ${\hat{p}}_{j}\left(i\right)$ from (2). Next, we rotate the shifted coordinates to fit the position of the nodes in A. In order to achieve this, we estimate a rotation angle, θ, which minimizes the errors between the evaluated locations of the nodes in A and their relative locations, as estimated directly by the assisting node. The evaluation of θ is performed by

(5a) $$\begin{array}{cc} \theta^{*}=\; & F\left\{\varepsilon (a)\right\}\; \end{array}$$

(5b) $$\begin{array}{cc} s.t.\;\;\; & \varepsilon \left(a\right)=\left\{{∥\left[\begin{array}{c} \cos\theta \;-\sin\theta \\ \sin\theta \;\;\;\;\cos\theta \end{array}\right]\;\left[\begin{array}{c} {\hat{p}}_{a}^{s,x}\left(i\right)\\ {\hat{p}}_{a}^{s,y}\left(i\right) \end{array}\right]-\left[\begin{array}{c} p_{a}^{x}\left(i\right)\\ p_{a}^{y}\left(i\right) \end{array}\right]∥}_{2}\right\}\;, \end{array}$$

where $\left({\hat{p}}_{a}^{s,x}\left(i\right),{\hat{p}}_{a}^{s,y}\left(i\right)\right)$, and $\left(p_{a}^{x}\left(i\right),p_{a}^{y}\left(i\right)\right)$ are the *x* and *y* coordinates of the shifted evaluated solution (4), and the *x* and *y* coordinates known relative positions of the nodes in A, respectively.

We consider several options for function F:

- Summation: $F=\underset{\theta }{\mathrm{argmin}}\sum_{a\in A}\varepsilon \left(a\right)$.
- Minimize: $F=\underset{\theta }{\mathrm{argmin}}\varepsilon \left(a\right),\;\forall a\in A$.
- Average: replace (5a) with $\theta^{*}\left(a\right)=\arg_{\theta }\min\left\{\varepsilon (a)\right\}$, and set $\theta^{*}=\sum \theta^{*}\left(a\right)/\left|A\right|,$∀a∈A.

The ‘summation’ approach gives equal weight to all nodes in A, while the ‘minimize’ approach performs rotation considering only the node in A that leads to the minimal error. The ‘average’ approach simply averages all the rotation angles that best fit each of the nodes in A. Finally, each node j∈N is rotated by

(6) $$\left[\begin{array}{c} {\hat{p}}_{j}^{r,x}\left(i\right)\\ {\hat{p}}_{j}^{r,y}\left(i\right) \end{array}\right]=\left[\begin{array}{c} \cos\theta^{*}\;-\sin\theta^{*}\\ \sin\theta^{*}\;\;\;\;\cos\theta^{*} \end{array}\right]\;\left[\begin{array}{c} {\hat{p}}_{j}^{s,x}\left(i\right)\\ {\hat{p}}_{j}^{s,y}\left(i\right) \end{array}\right]\;.$$

When solving through direct optimization, we plug the solution ${\left[{\hat{p}}_{j}^{r,x}\left(i\right),{\hat{p}}_{j}^{r,y}\left(i\right)\right]}^{T}$ at the input to (1). Note that, if solving through the relaxed optimization approach, the solution of (6) is the final estimate.

### 4.3. SOS Propagation

Our process starts with the transmission of the SOS messages by the SOS source. Considering packet failures, we let the SOS source transmit *R* SOS packets. Since the network’s graph is expected to be sparse, there is a need to relay the SOS message between the divers/nodes. Respecting the need for a prompt response to the SOS call, we adopt the flooding approach, so that a node successfully receiving the SOS packet immediately relays it further. Similar to the SOS transmission, each relayed packet is transmitted *R* times. However, to limit packet collisions, a node continues flooding an incoming packet only if the packet has not already visited all of its one-hop neighbor nodes. The latter requires piggy-backing the list of receiving nodes on top of each forwarded packet. To further mitigate packet collisions, the *R* re-transmissions from each node *i* are spaced by a pre-determined backoff $\Delta_{i}$. These backoff values are known to all nodes and can be chosen to reduce collisions as discussed in [34].

**Figure 2:**
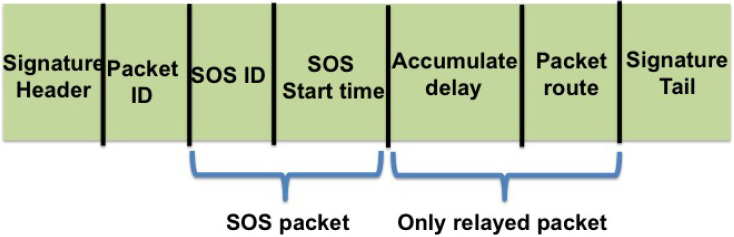
Structure of transmitted packets.

As illustrated in Figure 2, the relayed SOS packet includes information on the ID of the SOS source and the transmission time of the first SOS packet; the propagation time of the packet, considering the hardware/software delays in the relay node; and the packet’s route. While the latter is used to control the flooding procedure, together with the propagation time, it also gives an estimate of the time delay from the receiving node to the SOS source. This route information includes the ID of the relaying node as well as the number of the transmitted packets. For example, the route of a packet that passed through nodes s,n,p and *j* (in that order) is coded as $R_{j}=\left\{{\left[s,r_{s}\right]}^{T},{\left[n,r_{n}\right]}^{T},{\left[p,r_{p}\right]}^{T},{\left[j,r_{j}\right]}^{T}\right\}$ with two rows: $R_{j,1}$ and $R_{j,2}$, where, for a node $m\in R_{j,1}$, $r_{m}\in R_{j,2}$ is a scalar within the range {1,…,R} (recall *R* is the number of re-transmissions) determined by the number of re-transmissions of the same packet already made by node *m*. As we will show further on, the time delay and route information are also used to evaluate the time delay between the graph’s vertexes. To allow a separate time delay evaluation from each packet, the packets are numbered. Finally, considering the expected packet collisions, each packet is channel-coded and includes a cyclic redundancy check (CRC) to determine if it has been successfully received.

#### 4.3.1. Estimation of the Propagation Delay

The propagation delay in the link of an incoming SOS packet from a node *j* is determined by the packet’s transmission time and the measured ToF. To significantly reduce the packet’s size, rather than including the transmission time as part of the packet, we obtain the information on the propagation time in the link from node *j* based on the SOS transmission time, the accumulated time delay, and the route passed by the packet as explained in the following.

Let $T_{0}$ and $T_{i}$ be the SOS transmission time stamp (piggy-backed over the incoming packet, see Figure 2) and the time node *i* received the packet from *j*, respectively. In addition, denote $T_{j}^{d}$ as the accumulated delay up to node *j*, and let $T^{\mathrm{hardware}}$ be the known hardware time delay. The propagation delay between *i* and *j* is estimated by

(7) $$T_{i,j}^{\mathrm{pd}}=T_{i}-T_{j}^{d}-\sum_{n\in R_{j,1}}\left(r_{n}\cdot \Delta_{n}+T^{\mathrm{hardware}}\right)-T_{0}\;.$$

The time delay from node *i* to the SOS source *s* is upper bounded by

(8) $$T_{i,s}^{\mathrm{pd}}=T_{j}^{d}+T_{i,j}^{\mathrm{pd}}\;,$$

which is also coded in the outgoing packet from *i* as the new time delay value. That is, $T_{i}^{d}=T_{i,s}^{\mathrm{pd}}$.

Using (7), node *i* can form a graph of propagation delay information between any node pairs that appear in packets received by *i*. However, due to the many packet collisions, not all the packets may be received. As a result, some propagation delay information may be missing, and the delay graph would not be full. To complete the missing information, following [14], we run a shortest path algorithm (e.g., the Dijkstra algorithm) and obtain an upper bound for the missing delay by accumulating the delay along the shortest route. Let $M_{j,i}$ be the shortest pass route from node *j* to node *i*, arranged by connecting node pairs. The missing delay information is obtained by

(9) $$T_{i,j}^{\mathrm{pd}}=\sum_{\left(p,q\right)\in M_{j,i}}T_{p,q}^{\mathrm{pd}}\;.$$

Note that, in some cases, route $M_{j,i}$ cannot be found, for example, if no packet that passed through *j* was received by *i*. In this case, we eliminate node *j* from the graph constructed by *i*.

#### 4.3.2. SOS Decoding

Due to the flooding of the SOS packets, we expect many packet collisions. We observe that, although the flooded packets carry different information of the accumulated delay and routing path, the SOS information part is always the same. Considering this, we protect the SOS part of the packet by means of rateless coding [35]. In this way, although the decoding of single packets may fail, each incoming packet brings new information that is accumulated until the packet has been successfully decoded. To this end, we form a large coding matrix and allocate each node with columns to perform the rateless coding. To support the *R* re-transmissions made by each node, this list of columns allows each node to perform *R* rateless coding. That is, upon transmitting a packet, the transmitter codes by using its allocated columns from the coding matrix for the transmission of the *r* out of *R* similar packets.

By setting the above list of columns prior to the network’s deployment, we make sure it is known to all nodes, and the receiving node can add the received symbols to that already received from each transmitting node. Then, by identifying the IDs of the nodes transmitting the received packets, the receiver tries to decode the accumulated symbols using the columns of the coding matrix allocated to these transmitting nodes. As illustrated in Figure 2, to determine the ID of the transmitting nodes, and to identify the transmission number from the *R* possible ones, we include a signature sequence at the head and tail of each packet. This sequence is unique for each transmitter and for each of its *R* re-transmissions. Assuming only two packets collide at any given moment, at least one of the packets’ heads and tails is properly received, thereby allowing the identification of the transmission properties.

#### 4.3.3. ToA Estimation

Measuring the ToA, $T_{i}$, is trivial when the packet is properly decoded. We can also match $T_{i}$ and the transmitting node and set it according to the arrival of the head sequence, even when the packet fails. However, using rateless coding, we can also identify $T_{i}$ when the beginning of the packet collides with another packet. This is possible when the tail sequence has been properly decoded, such that the origin of the packet is identified. If the decoding fails and the beginning of the packet is corrupted, we identify the ToA based on the erasure symbols. In particular, we determine the arrival time as the beginning of a sequence of such erasure symbols.

Erasures are symbols identified by the process of rateless decoding as erroneous [35] for those symbols whose log-likelihood ratio (LLR) is low. Identifying the time instance when the two packets collide is performed in three steps:

1. **Smoothing:** Removing erasure symbols which do not appear as a sequence. In the absence of information about the distribution function of the symbols’ LLR, the smoothing operation is performed using a median filter of degree 3.
2. **Thresholding:** In the case where after the smoothing of individual erasures there exists only one sequence of erasures, we set the arrival time according to the sequence’s beginning time before smoothing. Otherwise, we further remove sequences of small numbers of erasures by setting a threshold on the number of erasure symbols. The threshold is set according to the expected symbol error rate (SER) and the measured signal-to-noise ratio (SNR). If an SNR measurement is not available (for example, when a commercial underwater modem is used), the SER is set empirically while removing the number of erasure symbols due to packet collisions. That is,
  (10) $$SER=\frac{S^{\mathrm{erasure},\mathrm{smooth}}}{2S^{\mathrm{packet}}-S^{\mathrm{erasure}}}\;,$$
  where $S^{\mathrm{erasure},\mathrm{smooth}}$ is the number of smoothed erasures, $S^{\mathrm{erasure}}$ is the number of erasure symbols after the smoothing procedure, and $S^{\mathrm{packet}}$ is the number of transmitted symbols in a packet. We then set a threshold, Th, to detect a sequence such that
  (11) $${\left(SER\right)}^{\mathrm{Th}}<\beta \;,$$
  where β is a user-defined probability to receive consecutive Th erasure symbols. We then identify the beginning of the packet collisions and the time instance when the colliding packet arrived as the start of the first sequence of at least Th consecutive erasure symbols.

## 5. Performance Evaluation

We will now discuss the performance of our SOS localization algorithm. We measure the performance in terms of the algorithm’s convergence time, $\rho_{\mathrm{converge}}$, and the Euclidean error of the evaluated SOS source location, $\rho_{\mathrm{error}}$. The convergence time is defined as the time elapsed between the transmission of the first SOS packet and the time until the last assisting node is able to estimate the location of the SOS source. To evaluate the performance of our algorithm, we conducted both numerical simulations and tests using underwater modems. The simulations explore the performance for various graph constellations, while tests in a water tank and in the field demonstrate the feasibility of the algorithm for use with real hardware and under harsh conditions.

### 5.1. Numerical Simulations

Our simulations involve five nodes, which is an appropriate number for divers performing maintenance work. Results are expected to improve as the number of nodes increases. We perform a Monte Carlo set of 1000 random placements of nodes and obstacles. In each run, the nodes are randomly placed in a square area of 2000 × 2000 m^2^. The communication range is set for 1500 m such that the graph is not fully connected, but no node is entirely disconnected. To further increase the graph’s sparsity, the square area includes horizontal and vertical obstacles, uniformly randomly placed in each simulation run with lengths uniformly distributed at [50,100] m, such that communication is possible only if an acoustic line-of-sight exists between the transmitter and the receiver. We consider the case of one and two nodes in A, whose relative locations are evaluated by the assisting node through range and bearing measurements. In each simulation run, we choose the identity of the nodes in A uniformly randomly.

We compare the performance of five methods: (1) our solution to (1) referred to as *ULB*; (2) our solution to (2) referred to as *UB*; (3) the non-classical MDS algorithm that allows missing links in the graph by an iterative solution starting from a random location; (4) a scheme presented in [21] termed *RBC-MDS*; and (5) a scheme presented in [22] termed *RPCA-MDS*. We choose the RBC-MDS as a benchmark since, like our approach, it handles missing links in a GL solution. Complementing this is the RPCA-MDS approach, which also utilizes partial information in the graph. Naturally, all schemes receive the same information regarding the links’ delays. The simulation results are analyzed by a cumulative density function (CDF). To obtain sufficient statistics, the 1000 simulated placements are divided into 10 points in the CDF.

Each simulation run starts with the transmission of an SOS packet of total duration 100 ms, including a synchronization signal of duration 50 ms. The SOS packet includes 32 bits and is protected by a (4,7) Reed–Solomon code, while relayed packets include 40 bits, further coded using a (5,7) Reed–Solomon code. Both packets include a CRC code of 9 bits. We consider a worst case scenario, where the corresponding symbols of all colliding packets are corrupted.

**Figure 3:**
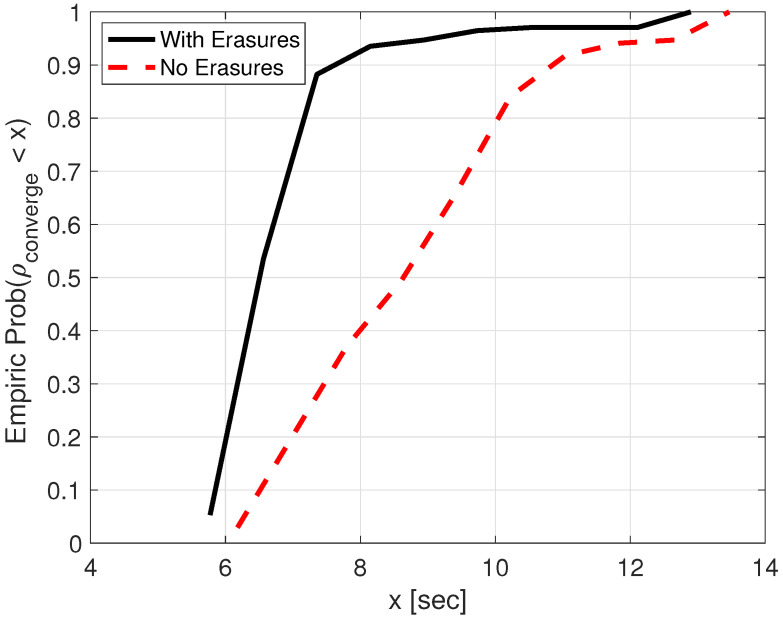
Empirical CDF of convergence time $\rho_{\mathrm{converge}}$. The average result is 8.5 s and 10.4 s for using and not using erasure information, respectively.

We start by exploring the delay in propagating the SOS packet across the divers’ network. In Figure 3, we show the CDF of the convergence time $\rho_{\mathrm{converge}}$. The results obtained are less than 10 s in 95% of the cases, and are quite robust, regardless of the graph topology. These results demonstrate the efficiency of our algorithm in rapidly flooding the SOS packet across the divers’ network, even in the presence of strong interference. Figure 3 also includes results when no symbol erasure information is used. The results, termed *No-Erasures*, show a significant degradation in performance compared to using erasures in the process of estimating the time-of-flight. This is because many packets collide when the SOS packet is flooded; however, using erasure indicators, we can still also evaluate the time-of-flight for these colliding packets.

**Figure 4:**
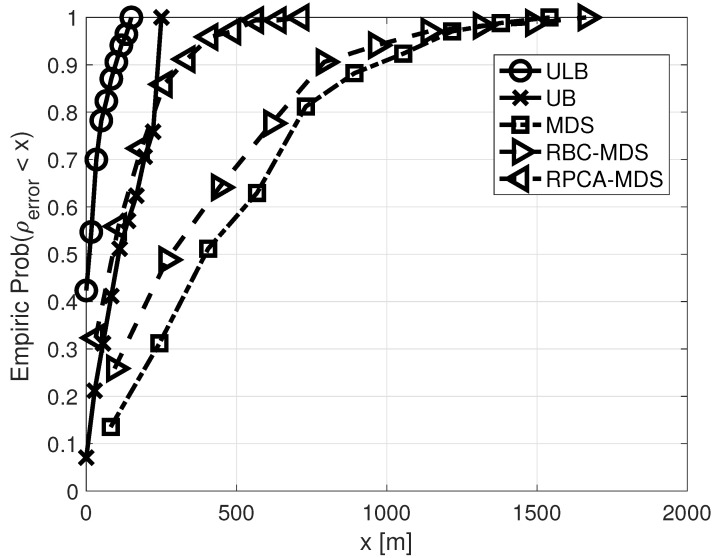
Empirical CDF of $\rho_{\mathrm{error}}$. Set A holds only one node.

Next, we consider the performance in terms of the Euclidean error, $\rho_{\mathrm{error}}$. The CDF of $\rho_{\mathrm{error}}$ is shown in Figure 4 when set A holds only a single node. We report that the average result for the use of both upper and lower bounds is 36.4 m; the average result for the use of only upper bounds is 160.9 m; the average result for MDS is 542.5 m; the average result for RBC-MDS is 442.6; and the average result for RPCA-MDS is 155.9. We observe that, due to its use of both direct and partial information, the results of RPCA-MDS are better than those of MDS and RBC-MDS, and are somewhat similar to UB. However, the improved results of ULB demonstrate the clear advantage of using conditional localization for the task of SOS localization. Observing the small variation of the results observed for conditional localization, we conclude that this method is robust in terms of the graph topology. Comparing the results of both types of conditional localization, a clear advantage of using both the upper and lower bounds is observed, at the cost of complexity.

**Figure 5:**
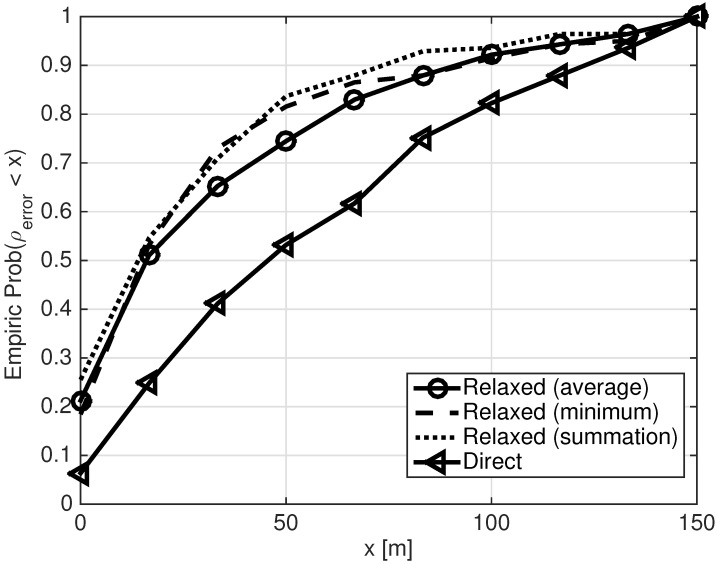
Empirical CDF of $\rho_{\mathrm{error}}$ for the solution of (1), and for the solution of (2) using the three rotation options for setting F in (5a). Set A includes two nodes.

To comment on the proper way to localize the SOS source when set A holds two nodes of estimated relative locations, in Figure 5, we show the CDF of $\rho_{\mathrm{error}}$ for the solution of the direct optimization problem (1) (*Direct*), and for the solution of the relaxed optimization problem (2) (*Relaxed*) using the three rotation options described in Section 4.2.2. We conclude that the Relaxed solution is always better than the Direct one. This is because the performance of the Direct solution relies on the performance of the rotated MDS solution, which-as observed by the results of Figure 4—is, in fact, inaccurate. Comparing the three options to define (5a), we observe that the performance of the ‘Summation’ approach is far better than that of the ‘Average’ approach, and slightly better than the ‘Minimize’ approach. This is mainly because the ‘Summation’ approach accounts for all nodes in A, and we used accurate relative locations of the nodes in A.

**Figure 6:**
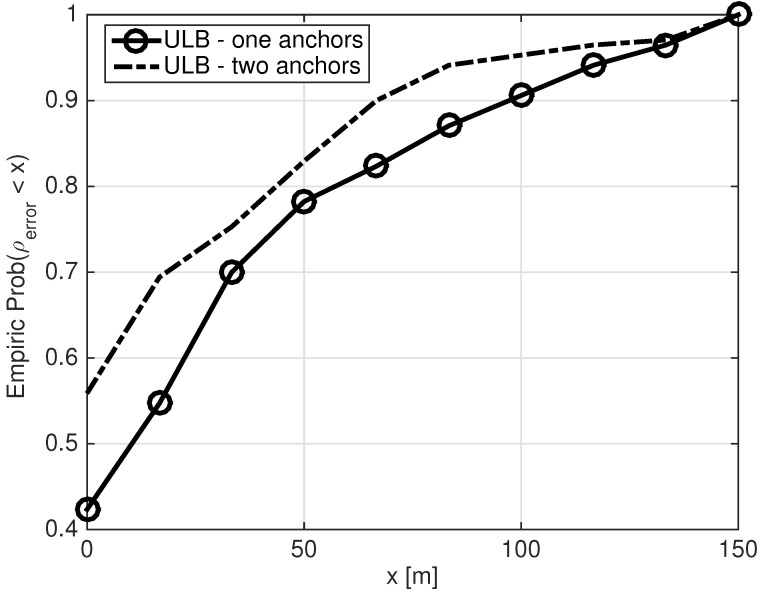
Empirical CDF of $\rho_{\mathrm{error}}$. Set A holds one and two nodes. F in (5a) is defined according to the ‘Summation’ approach.

We now compare the performance of the ULB conditional localization method when set A holds one or two nodes. The results are presented in Figure 6 in terms of the CDF of $\rho_{\mathrm{error}}$. As expected, the results improve when set A includes two nodes, with an average gain of 11.3 m. However, this requires the assisting node to know its location, relative to two nodes, which is not always guaranteed. Still, observing the absolute Euclidean error, we conclude that the ULB method performs well and provides the localization capability of a diver in distress with sufficient accuracy when set A includes only one node.

### 5.2. Tests in Water Tank

**Figure 7:**
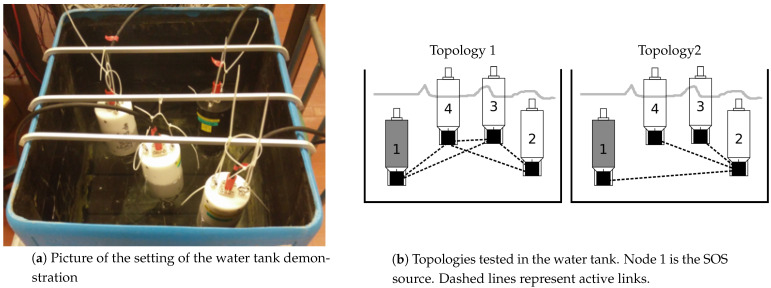
Tank experiment.

To test the feasibility of our algorithm with real hardware, we set up a proof-of-concept test in a water tank of size 60 × 70 cm. To reduce reverberations, the walls and floor of the tank were covered with sound-absorbing material. Using the T2Mo tool [36], we accessed the physical layer of four EvoLogics modems to employ our algorithm. As is depicted in Figure 7a, the black modem was set as the SOS source. While the modems were stationed very close to each other, due to the strong interference in the tank, the formed network graph was sparse. In particular, as illustrated in Figure 7b, two topologies were tested, and five runs were performed for each topology.

Since the tank dimensions are small, performance is only measured in terms of the delay until the SOS information is received (*SOS detection*), and in terms of the delay until a node receives enough information to perform localization of the SOS source, i.e., the algorithm convergence time (*Data collection*). The results are presented in Table 2. We observe quite small delays. In fact, the main delays are due to the modems’ processing time, and to packet collisions. The mean delay for having the SOS information is 3.25 s, and the average convergence time is 24.53 s. Due to the long propagation delay in the acoustic link, these delays are expected to grow for real underwater channels; at the same time, less collisions are expected. This was demonstrated using data from a sea experiment, as discussed in the following.

### 5.3. Data from a Sea Experiment

**Figure 8:**
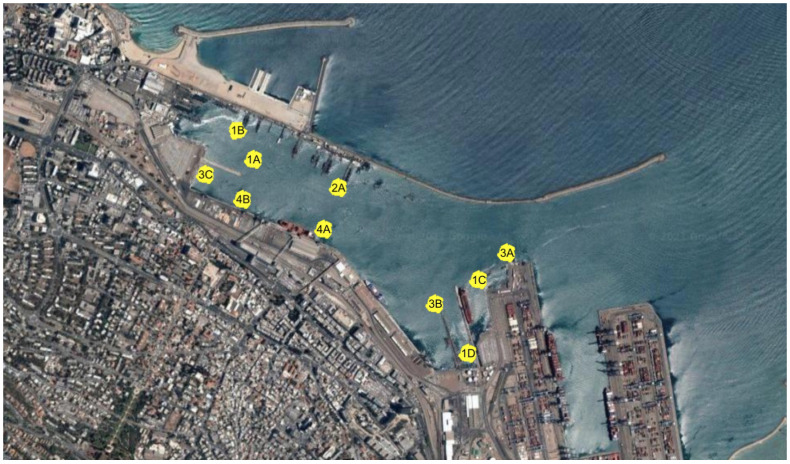
Map of the sea experiment (picture taken from Google Maps).

To demonstrate the performance of our approach in realistic scenarios, we examined playback results from a sea experiment performed in May 2009 in the Haifa harbor, Israel, for an underwater acoustic network formed by four boats creating five different sparse network topologies, as illustrated in Figure 8. As described in [37], the playback is performed by sending the SOS packet and its relays through the channels recorded in the experiment, and by considering the recorded delays in the nodes and in the communication links. Using this type of data-augmented approach, we take into account both real delays in the channel and distortions caused by the ambient noise and the multipath channel. The data from this experiment are shared in [38].

We test performance in terms of the delay from an SOS transmission until convergence of the assisting nodes, and by the range and bearing accuracy averaged for the choice of the SOS source. The results of ULB are presented in Table 3 and are compared against the MDS, RBC-MDS, and RPCA-MDS benchmarks. We observe that, mostly because no limitations are set for MDS, MDS has a slight advantage over the other schemes in terms of the convergence time, with an average convergence time of 11.6 s vs. 12.5 s for our ULB scheme. In addition, for the fully connected network in Topology 1, MDS obtains the best performance. However, similar to the simulation results, when the topology is sparse, MDS performance degrades significantly compared to the other approaches, while the performance of RPCA-MDS far exceeds it. Still, in terms of localization accuracy, for all sparse topologies, ULB achieves the best performance, with an average localization error across topologies of 44.8 m. We note that these results of CGL are of the same order as those in our simulations, which reflects CGL’s ability to also perform well in realistic channels.

## 6. Conclusions

In this paper, we suggested an efficient distributed GL algorithm for estimating the relative location of a diver in distress, to enable timely assistance provided by fellow divers. For each possible assisting diver, our GL uses propagation delay measurements from the received or forwarded SOS packets, and solves an optimization problem for the projection of inter-divers’ distances into locations, while constraining the solution by upper and lower bounding the diver-to-diver distances. Using the location of the assisting diver as a pivot, we shift and rotate the resulting position of the SOS source to find its location, relative to that of the assisting node. Our approach does not require the use of any anchor nodes of known locations, and requires the direct measurement of only bearing and range to one single node in the network. Considering the expected sparsity of the divers’ network graph, we flood the SOS packet to quickly propagate the SOS information, and use rateless coding to combat packet collisions. These collisions are, in fact, utilized to determine the time-of-flight of packets according to the start of a sequence of erroneous symbols. Numerical simulations show that, without using any anchor nodes, our method evaluates the location of the SOS source with an accuracy of the order of a few meters. Results from a controlled experiment in a water tank, and data used from a sea experiment demonstrate the system’s capability to localize a diver in distress in a real environment using actual underwater modems. Future research will include the investigation of further GL algorithms.

## Figures and Tables

**Table 1:** List of major notations.

Notations	Explanations
N	Set of nodes in the network
*s*	ID of diver in distress
A	Nodes whose location relative to the assisting node is known
$p_{j}\left(i\right)$	Location of node *j* relative to that of node *i*
$M_{n,m}$	shortest pass route from node *n* to node *m*
$U_{n,m},\;L_{n,m}$	Upper and lower bound in link (n,m), respectively.
$T_{n,m}^{\mathrm{pd}}$	Propagation delay in link (n,m)
$R_{j}$	Route of a packet passed from a source to a node *j*
Λ	Maximum error of measured inter-node distances
*R*	Number of allowed re-transmissions of the same packet

**Table 2:** Tank test results.

		Node 2	Node 3	Node 4
Topology 1	SOS detection (s)	2.147	2.356	0.6
	Data collection (s)	26.38	30.08	25.76
Topology 2	SOS detection (s)	0.35	5.24	9.76
	Data collection (s)	25.16	19.91	19.89

**Table 3:** Sea experiment results for playback data from [38]. Five topologies are analyzed.

Topology	Metric	CGL (ULB)	MDS	RBC-MDS	RPCA-MDS
Topology 1	Delay (s)	7.8	7.5	7.7	7.9
	Accuracy [m]	16	13	15	16
Topology 2	Delay (s)	11.3	10.8	11	11.5
	Accuracy [m]	22	98	45	32
Topology 3	Delay (s)	9.2	8.6	8.9	9.2
	Accuracy [m]	45	173	61	56
Topology 4	Delay (s)	16.1	14.6	15.2	16.3
	Accuracy [m]	63	221	87	73
Topology 5	Delay (s)	18.2	16.5	17.5	18.2
	Accuracy [m]	78	215	117	101
